# Surface Quality Enhancement of SLM-Fabricated Ti-6Al-4V via Top-Hat Laser Polishing: Melt Pool Dynamics and Microstructural Evolution

**DOI:** 10.3390/nano16090505

**Published:** 2026-04-22

**Authors:** Yingwei Kuang, Mingjun Liu, Haibing Xiao, Zhenmin Wang, Bowie Luo, Xiaomei Xu, Shun Gu

**Affiliations:** 1 School of Intelligent Manufacturing and Equipment, Shenzhen University of Information Technology, Shenzhen 518172, China; 2 School of Mechanical and Automotive Engineering, South China University of Technology, Guangzhou 510641, China; 3 Han’s Laser Technology Industry Group Co., Ltd., Shenzhen 518052, China

**Keywords:** selective laser melting, top-hat laser polishing, titanium alloy, surface roughness reduction, microstructure

## Abstract

Ti-6Al-4V parts fabricated via selective laser melting (SLM) often exhibit severe surface irregularities that limit their direct engineering application. This study proposes a top-hat beam laser polishing method to improve surface quality. The results show that surface roughness (Sa) is reduced to 0.48 μm, a 95.3% decrease from the as-built condition. The uniform energy distribution of the top-hat beam stabilizes melt pool behavior, enabling effective surface leveling through valley filling and lateral melt flow. In contrast, Gaussian beam polishing induces strong Marangoni convection and wake effects, resulting in higher residual roughness. Microstructural analysis indicates an increased fraction of equiaxed α grains and a β-phase content of ~6% after top-hat polishing. The heat-affected zone likely exhibits a subcritical heat-treatment-like effect, promoting fine secondary α precipitation. Additionally, localized stresses induced by steep thermal gradients during SLM are effectively relieved. Overall, top-hat laser polishing is a promising post-processing technique for enhancing the surface quality of Ti-6Al-4V components.

## 1. Introduction

Ti-6Al-4V, a typical α + β titanium alloy, is widely used in aerospace, automotive, and biomedical fields due to its high strength-to-weight ratio, fracture resistance, corrosion resistance, and biocompatibility [1]. However, its low thermal conductivity and high reactivity at elevated temperatures hinder fabrication, while conventional methods such as forging are costly and time-consuming [2]. Additive manufacturing (AM), particularly powder-bed fusion techniques such as selective laser melting (SLM) and electron beam melting, provides an efficient approach for producing complex titanium components [3,4]. Despite these advantages, powder-based AM parts typically exhibit high surface roughness [5], often exceeding 10 μm, which fails to meet the stringent requirements of high-precision applications such as aerospace and medical devices [6]. These applications generally require surface roughness below 1 μm to reduce stress concentrations and suppress fatigue crack initiation [7]. Consequently, as-built SLM components are often unsuitable for high-stress environments without post-processing, leading to reduced service performance and lifetime [8,9]. Conventional post-processing methods, including sandblasting, mechanical polishing, and electrochemical polishing, are limited by low efficiency, environmental concerns, and inadequate precision for microscale refinement [10]. In contrast, laser polishing offers a non-contact and efficient alternative for surface improvement [11]. This process smooths the surface by selectively melting a thin layer, with molten material redistributing from peaks to valleys, primarily driven by surface tension and gravity [12].

Experimental studies have confirmed that laser polishing effectively improves surface finish across various materials. Jaritngam et al. [13] reduced the surface roughness of a titanium alloy by 43%, while Zhang et al. [14] reported a 62.3% reduction for SLM-fabricated Ti-6Al-4V. Similarly, Ma et al. [15] demonstrated that laser processing could decrease surface roughness from over 5 µm to below 1 µm. The effectiveness of laser polishing is strongly influenced by beam shaping. Wang et al. [16] used a top-hat beam in CO_2_ laser smoothing of fused silica, reducing roughness from ~500 nm to below 0.5 nm. Tian et al. [17] reported that laser polishing of EBM-fabricated Ti-6Al-4V reduced roughness to below 0.51 μm while also causing grain refinement, phase transformation, and increased subsurface hardness. Liu et al. [18] further investigated polishing efficiency and hardness reduction mechanisms in martensitic mold steel using a nanosecond top-hat beam. Computational modeling has also played an important role in understanding laser polishing mechanisms. Pham et al. [19] used multi-physics simulations to predict the surface roughness of laser-polished SS316L. Wang [20] analyzed the influence of capillary and thermocapillary forces on bulge formation during micro-melting and solidification of 304 stainless steel. For Ti-6Al-4V, Li et al. [21] developed a 2D model to simulate surface evolution and melt pool dynamics under a top-hat laser beam. Marimuthu et al. [22] combined simulations and experiments to polish SLM Ti-6Al-4V, reducing surface roughness from 10.2 μm to 2.4 μm, demonstrating that beam shaping can further optimize surface topography.

Despite recent advances, two critical gaps remain in the literature. First, most studies on laser polishing of SLM-fabricated Ti-6Al-4V focus on conventional Gaussian beams, while systematic investigations of top-hat beam profiles at the microscale are limited. Consequently, the benefits of uniform energy distribution for enhancing polishing efficiency and surface uniformity remain poorly quantified. Second, although surface roughness reduction is widely reported, the interplay between beam profile, melt pool dynamics, and subsurface microstructural evolution is not well understood. In particular, the mechanisms governing refined microstructures and secondary phase formation during rapid remelting and solidification remain unclear, despite their importance for fatigue performance and structural reliability.

To address these gaps, this study systematically links beam shaping, surface morphology evolution, and subsurface microstructural transformation in SLM Ti-6Al-4V. A direct comparison between top-hat and Gaussian beam polishing under controlled conditions is conducted, revealing melt pool flow and thermal field characteristics unique to top-hat irradiation through combined numerical simulations and experiments. Additionally, the formation of an equiaxed grain-dominated surface microstructure and secondary phase evolution in the heat-affected zone provides new insight into the relationship between laser polishing and microstructural evolution.

The results demonstrate that enlarging the beam diameter to the sub-millimeter scale effectively reduces localized energy concentration, thereby enhancing polishing uniformity and efficiency. Moreover, the top-hat beam induces a more balanced thermal cycle, producing an effect analogous to in situ heat treatment, which promotes microstructural homogenization. This work not only advances the fundamental understanding of beam shaping effects in laser polishing but also provides practical guidance for tailoring surface integrity and microstructural performance in additively manufactured Ti-6Al-4V components.

## 2. Materials and Experimental Methods

### 2.1. Materials and Laser Polishing Process

**Figure 1:**
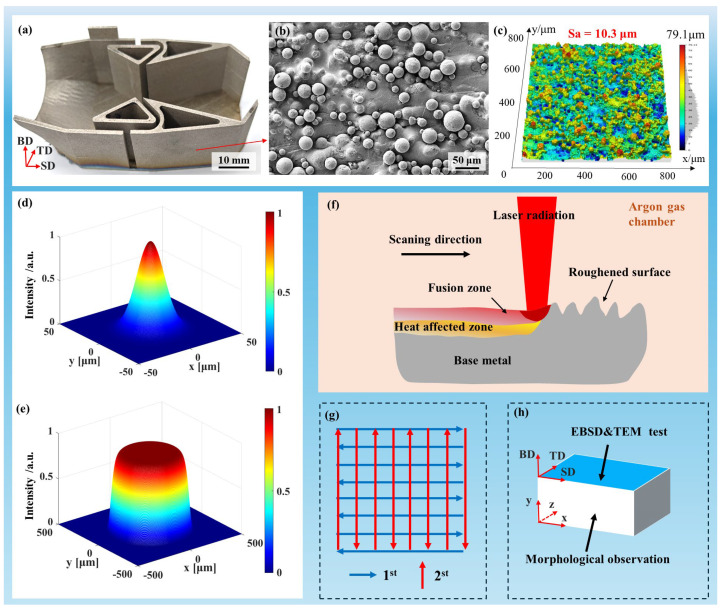
Experimental material and laser polishing setup. (**a**) As-fabricated thin-walled SLM component. (**b**,**c**) Surface topography and roughness of the as-built specimen. (**d**,**e**) Gaussian and top-hat beam intensity distributions. (**f**) Schematic of laser polishing setup. (**g**) Bow-shaped scanning path. (**h**) Sample configuration for surface and microstructure characterization.

**Figure 2:**
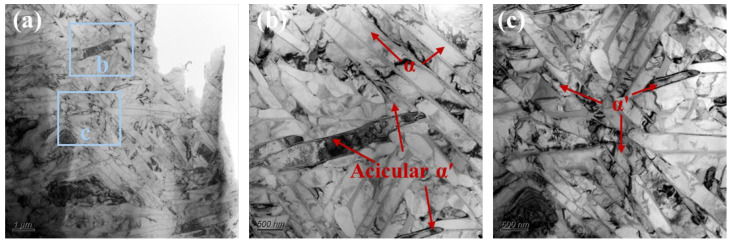
The microstructure of the base metal: (**a**) low-magnification image; (**b**,**c**) higher-magnification views of selected regions.

The samples used for laser polishing were complex thin-walled Ti-6Al-4V structures with an average wall thickness of 1.2 mm, fabricated via selective laser melting using a HANS-100 system (Han’s Laser, Shenzhen, China). Gas-atomized Ti-6Al-4V pre-alloyed powder with particle sizes of 25–50 μm was used under optimized SLM processing conditions. The three orthogonal directions were defined as the scanning (SD), transverse (TD), and build (BD) directions. Figure 1a shows the high roughness of the as-built surface, where the surface features are comparable to the powder particle size. As shown in Figure 1b, this morphology mainly results from partially melted powder particles adhered to the surface. The initial areal roughness (Sa) was measured to be 10.3 μm (Figure 1c). In addition, the as-built thin-wall microstructure (Figure 2) primarily consists of α′ and α phases.

To compare different laser polishing processes, experiments were performed using two beam profiles. A Gaussian beam, generated by an infrared fiber laser (QCW100, Han’s Laser, Shenzhen, China) at 1080 nm, delivered up to 100 W in continuous-wave mode with a focused spot diameter of ~50 μm (Figure 1d). In contrast, a top-hat beam, produced by a DK-YSM 550 fiber laser (DK Laser, Yueyang, China) at 1070 nm, provided a more uniform energy distribution, larger spot size (~600 μm), and higher divergence (Figure 1e), with a maximum power of 550 W.

The beam profile has a significant effect on the distribution of energy across the surface, which directly influences the thermal gradients and the material response during the polishing process. The Gaussian beam generates a concentrated energy distribution, resulting in more localized heating, which may lead to different melt pool dynamics compared to the top-hat beam, which provides a more uniform energy distribution across the spot. This difference in energy deposition plays a primary role in the observed differences in surface quality and microstructural evolution, which we attribute mainly to the beam shape.

Figure 1f illustrates the laser polishing setup. To minimize oxidation, experiments were conducted in an enclosed chamber under an argon atmosphere. A two-dimensional galvanometer scanner directed the focused laser beam onto the sample surface, inducing localized melting. The irradiated region consists of two zones: the fusion zone (FZ) and the heat-affected zone (HAZ). As shown in Figure 1g, two consecutive polishing passes were applied along a bow-shaped scanning path.

Critical processing parameters, such as laser power density and scanning velocity, were systematically optimized for both Gaussian and top-hat beam polishing conditions. Given the use of a continuous-wave laser, the incident energy was characterized by the laser power density (E), as derived from the following equation:


(1) $$E=\frac{4P}{\pi D^{2}}$$

In this expression, P corresponds to the laser’s average output power, and D signifies the diameter of the focal spot.

Table 1 presents the complete set of processing parameters employed for both polishing approaches. The quality of the polished surface was primarily determined by the laser power density and the scanning speed. The beam profile plays a critical role in governing the spatial distribution of energy and the resulting thermal gradients (Table S1). The investigated ranges were 1528–4586 kW/cm^2^ for the Gaussian laser and 71–176 kW/cm^2^ for the top-hat laser, while the scanning speeds varied from 100 to 1500 mm/s. Based on the power range of the laser equipment used, the laser power range listed in the table was selected for this study. The range of laser power density was subsequently determined according to Equation (1). Laser scanning speed, as a key influencing factor, was determined through preliminary experiments to determine the maximum and minimum scanning speeds. A bow-shaped scanning path was employed for all tests. Regarding the number of spot overlaps, previous research has shown that the number of surface scanning passes has a limited effect on surface quality [18]. To ensure uniform scanning tracks in both directions, each sample underwent two polishing passes: the first pass with the scanning direction parallel to the SD, and the second pass parallel to the BD. Regarding the overlap ratio, studies have adopted a spot track overlap of approximately 90% [8]; therefore, a fixed track offset and overlap rate were determined based on the laser spot diameter.

### 2.2. Surface Morphology and Microstructure Characterization

After laser polishing, the surface morphology and microstructure of SLM-fabricated Ti-6Al-4V thin-walled parts were systematically analyzed. Surface features were observed using an optical microscope (DMI3000 M, Leica, Wetzlar, Germany), and areal roughness parameters (Sa, Ra(x), Ra(y)) were measured with a 3D confocal microscope (MarSurf CM mobile, Mahr, Augsburg, Germany) over an 800 μm × 800 μm area with 800 μm line scans in both directions. Each sample was measured in three regions for reproducibility, using a 20× objective (M1042, Mahr, Augsburg, Germany). Data acquisition and processing were performed with MarSurf Metrology and MarSurf MfM Premium software 8.1.

EBSD mapping on the x–z plane was conducted using a Gemini 300 SEM (ZEISS, Oberkochen, Germany) with a 0.2 µm step size, and datasets were processed in Channel 5 software. Site-specific thin-foil specimens from the FZ, HAZ, and BM were prepared via ion milling and examined via TEM (Talos F200X, FEI, Hillsboro, OR, USA).

## 3. Numerical Simulations

Melt pool temperature and flow during laser polishing were analyzed using a two-dimensional COMSOL Multiphysics model that couples heat transfer and fluid flow, allowing comparison between Gaussian and top-hat beam processes. The model assumptions and governing equations are summarized in this section. To balance computational efficiency and accuracy, the melt pool was treated as an incompressible, Newtonian, laminar fluid, and the material was assumed to be isotropic and homogeneous. Plasma formation and significant vaporization were considered negligible, and recoil pressure was neglected for both beam configurations. Evaporation was included only as a latent heat loss term in the surface energy balance, while mass loss, vapor flow, and recoil pressure effects were not considered.

### 3.1. Simulation Model and Material Properties

**Figure 3:**
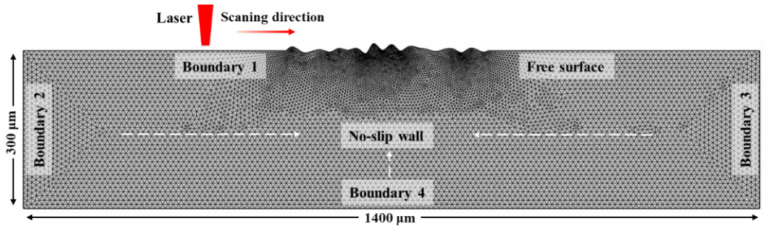
Schematic representation and meshing of computational domain used for numerical modeling.

The simulation domain for both laser polishing configurations measured 300 μm × 1400 μm (Figure 3). An initial non-planar surface profile was applied to represent the as-built Ti-6Al-4V topography, including typical peaks and valleys. A locally refined mesh was used near the surface to resolve steep temperature gradients induced by the laser. An average mesh size of 3 μm and an output time interval of 0.01 ms were used in the simulations, with these parameters selected based on the analyses presented in Figures S1–S3 and Tables S2 and S3. The thermophysical properties of Ti-6Al-4V as a function of temperature are listed in Table 2. The laser power density and scanning speed in the model were based on optimal experimental polishing conditions (Table 3).

### 3.2. Governing Equations

The laser polishing temperature field simulation model belongs to a nonlinear transient Ti-6Al-4V conduction model, in which the differential equation of thermal conductivity is expressed as Equation (2), the system’s mass and momentum conservation laws are formulated in Equations (3) and (4) [23]:


(2) $$\rho C\frac{\partial T}{\partial t}+\rho C\nabla \cdot \left(\vec{u}T\right)-\nabla \cdot (k\nabla T)=0$$

(3) $$\rho \nabla \cdot \vec{u}=0$$

(4) $$\rho \frac{\partial u}{\partial t}+\rho \vec{u}\cdot \left(\nabla \vec{u}\right)=\nabla \cdot [-pI+\mu (\nabla \vec{u})+\left(\nabla^{T}\vec{u}\right)]+{\vec{F}}_{v}$$

Here, the symbol ρ refers to alloy density, C is the constant-pressure specific heat, T represents the time-dependent temperature field, t represents time, and k indicates thermal conductivity. Additionally, p defines the melt pool pressure, μ stands for the dynamic viscosity, I denotes the identity tensor, and ${\vec{F}}_{v}$ represents the body-force source term determined by the boundary conditions [23].


(5) $${\vec{F}}_{v}={\vec{F}}_{g}+{\vec{F}}_{b}=\rho \left[1-\beta \left(T-T_{m}\right)\right]g$$

To account for the latent heat associated with melting, an effective heat capacity, denoted as $C^{*}$, is introduced and defined according to the following expression:


(6) $$C^{*}=C+L_{m}\frac{{df}_{l}}{dT}$$

Here, $L_{m}$ corresponds to the latent heat of fusion.

To promote computational robustness during phase change and facilitate a continuous transition from solid to liquid, the liquidus temperature ($T_{l}$) was assigned a value marginally higher than the equilibrium melting point, with the solidus temperature ($T_{s}$) positioned just below this threshold. The interval spanning $T_{s}$ to $T_{l}$ is identified as the mushy region. The material’s effective properties are controlled by the evolving phase fraction, expressed as the temperature-dependent liquid fraction ($f_{l}$).

Within the mushy region, $f_{l}$ is modeled as a linear function of temperature based on the methodology in the reference [24]:


(7) $$f_{l}=\{\begin{array}{c} 0\;T\leq T_{s}\\ \frac{T-T_{s}}{T_{l}-T_{s}}\;T_{s}\leq T\leq T_{l}\\ 1\;T\geq T_{l} \end{array}$$

### 3.3. Boundary Conditions

The 2D time-dependent model formulated in this work requires the definition of thermal and momentum boundary conditions for three key regions: the top surface subjected to laser radiation, the side boundaries, and the substrate. The configuration of these boundaries is illustrated in Figure 3.

The energy distribution of the continuous-wave laser spot follows a Gaussian profile, which can be mathematically expressed as [18]:


(8) $$E_{G}=\alpha \frac{2P}{\pi r_{0}^{2}}exp\left[\frac{{-2(r-vt)}^{2}}{r_{0}^{2}}\right]$$

The mathematical expression for the uniform energy distribution characterizing the top-hat laser spot is defined as follows [25]:

(9) $$E_{T}=\alpha \frac{P}{\pi r_{0}^{2}}y(r)$$

where α is the laser absorptivity, P is the applied average laser power, r is the distance from the current computational cell to the heating center, y(r) is the range of laser irradiation with the laser energy beyond the diameter of the laser spot considered zero, $r_{0}$ is the beam’s effective radius, and v  is the speed at which the laser spot moves.

Heat transfer boundary condition: At boundary 1 (top surface in Figure 3), the thermal behavior is described by Equations (10) and (11), which include contributions from laser input and heat dissipation by evaporation, convective flow, and radiative transfer. Thermal insulation is applied at boundary 4, as formulated in Equation (12).


(10) $$q_{radi}=\sigma \varepsilon (T^{4}-T_{0}^{4})$$

(11) $$q_{con}=h(T-T_{0})$$

(12) −k∇T=0

In these expressions, the parameters are as follows: σ represents the Stefan-Boltzmann constant, ε denotes the surface emissivity, and h denotes the convective heat transfer coefficient.

The momentum boundary condition is defined as follows. Equation (13) specifies a no-slip condition for the velocity field at boundaries 3 and 4. Additionally, the radial r velocity component is set to zero at these boundaries, as specified in Equation (14).


(13) $$u_{r}=u_{z}=0$$

(14) $$u_{r}=0,\frac{{\partial u}_{z}}{\partial r}=0$$

The velocity components of the fluid flow are denoted as $u_{r}$ (radial direction) and $\;u_{z}$ (axial direction), corresponding to the radial and axial coordinate axes, respectively.

Free surface boundary condition: For laser polishing, the momentum source term defined in Equation (4) acts directly at the molten free surface (boundary 1). Consequently, the irradiated zone is influenced by two primary competing flow mechanisms: the normal surface tension flow $\sigma_{n}$ induced by surface tension wave oscillations at the melt pool surface, and the tangential thermocapillary force (Marangoni effect) $\sigma_{t}$, which exhibits temperature gradient dependence [18].


(15) $$\sigma_{n}=[\gamma_{m}-A_{\gamma }(T-T_{m})]\kappa \cdot \vec{n}$$

(16) $$\sigma_{t}=\frac{\partial \gamma }{\partial T}\vec{\nabla }T\cdot \vec{t}$$

Here, κ refers to the free surface curvature, $\gamma_{m}$ indicates the surface tension of pure titanium, $A_{\gamma }\;$ defines its temperature-dependent variation. The term $\vec{\nabla }T$ denotes the gradient of temperature in the tangential direction, $\vec{n}\;$ represents the outward unit normal vector, and $\vec{t}$ indicates the unit tangent vector along the free surface.

Moving mesh boundary condition: Free surface movement was described using the Arbitrary Lagrangian–Eulerian (ALE) framework. In this framework, interior mesh nodes are governed by Eulerian principles, while nodes located on the domain boundary adhere to a Lagrangian description. This combined strategy ensures that boundary motion is explicitly tracked and governed by fluid dynamics. The following equation describes the coupled behavior of the ALE framework and the fluid flow.


(17) $$u_{mesh}\vec{n}=u_{mat}\vec{n}$$

In this expression, $u_{mesh}$ corresponds to the mesh motion velocity, whereas $u_{mat}$ describes the flow velocity of the molten fluid. Table 4 presents the boundary conditions imposed on the relevant physical fields.

## 4. Results and Discussion

### 4.1. Influence of Parameters on Surface Morphology

**Figure 4:**
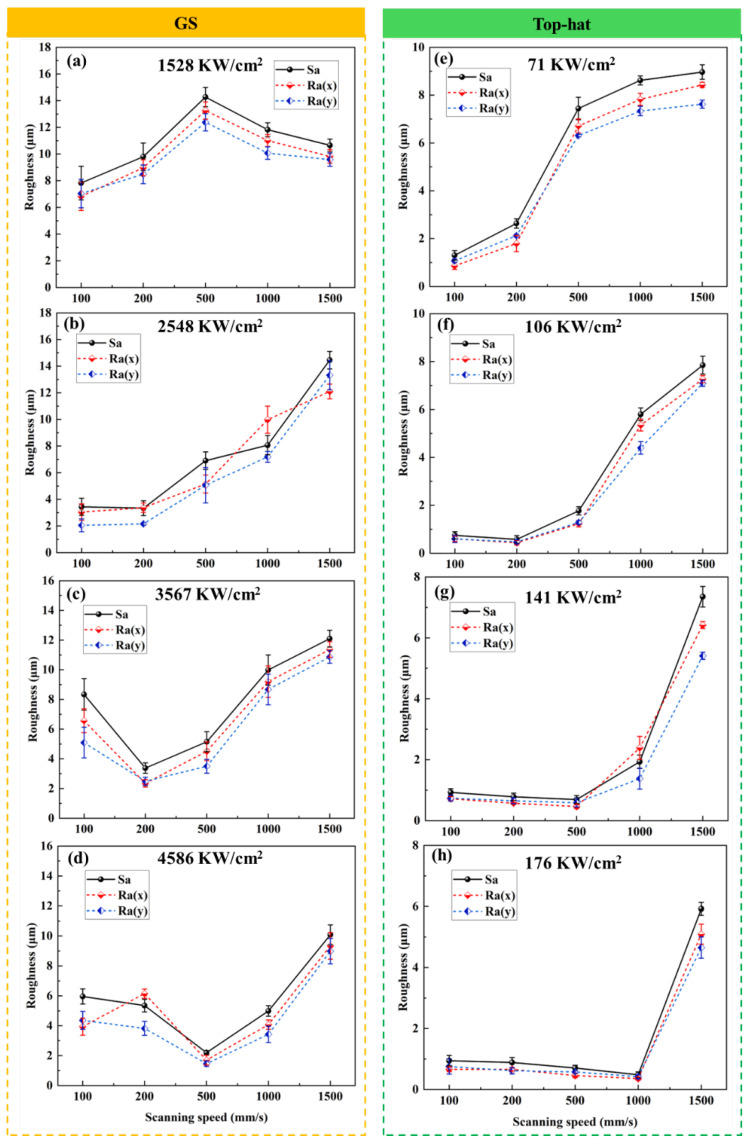
Influence of laser power density and scanning speed on surface roughness for (**a**–**d**) Gaussian and (**e**–**h**) Top-hat laser processes.

Surface roughness results for Gaussian laser polishing are shown in Figure 4a–d. At 1528 W/cm^2^, the polished surface remained rough, exceeding the as-built condition. Roughness decreased with increasing power density, reaching a minimum of 2.21 μm at 4586 W/cm^2^ and 500 mm/s, though the trend with scanning speed was non-monotonic. The top-hat laser polishing results are shown in Figure 4e–h. At 71 W/cm^2^, surface roughness was high but lower at slower scan speeds, where sufficient energy enabled complete remelting and surface leveling. Higher scan speeds delivered less energy, leaving surface asperities and increasing roughness [26,27]. Increasing power density generally reduced roughness; at 176 W/cm^2^, roughness varied parabolically with scan speed, reaching a minimum of 0.48 μm at 1000 mm/s, outperforming the optimal Gaussian outcome.

**Figure 5:**
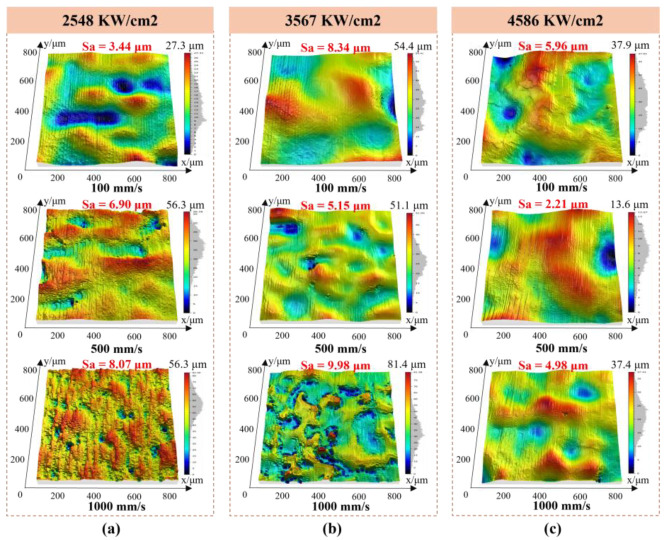
The surface 3D morphology of samples processed with Gaussian laser polishing at different parameters. The applied laser power densities were (**a**) 2548 W/cm^2^, (**b**) 3567 W/cm^2^, and (**c**) 4586 W/cm^2^, with scan speeds between 100 and 1000 mm/s.

Surface roughness evolution can be understood through surface morphology analysis. Figure 5 shows the 3D topography of Ti-6Al-4V after Gaussian laser polishing. At 2548 W/cm^2^ and 100 mm/s, pronounced remelting occurs, producing an undulating surface with prominent ridges and troughs. The low scan speed provides sufficient energy for complete melting, but the concentrated heat generates steep temperature gradients. The interaction of surface tension, Marangoni convection, and gravity amplifies internal melt flow, causing significant surface undulations [28]. At 4586 W/cm^2^ and 100 mm/s, increased melt pool turbulence further raises roughness. Conversely, a higher scan speed of 500 mm/s shortens the laser–material interaction, reduces melt pool agitation, and yields a smoother surface.

**Figure 6:**
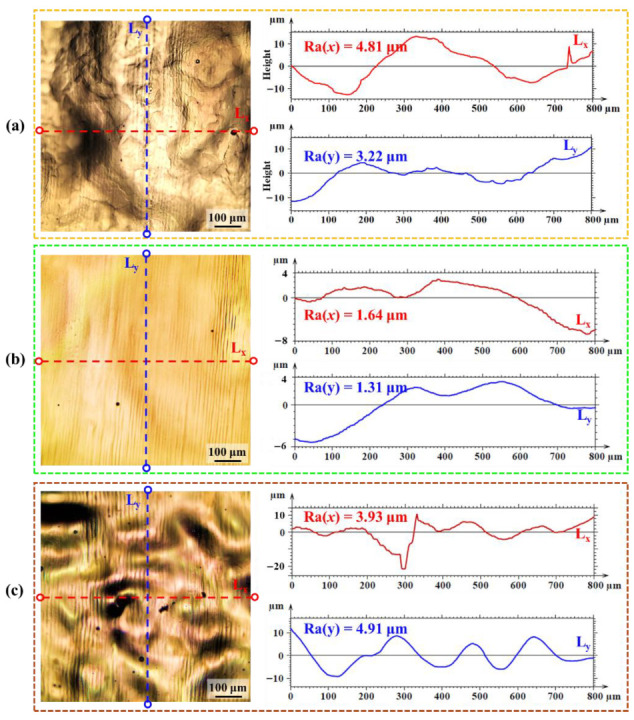
Surface morphology and orthogonal profile curves of specimens processed under representative Gaussian laser polishing parameters: (**a**) 4586 W/cm^2^–100 mm/s, (**b**) 4586 W/cm^2^–500 mm/s, (**c**) 3567 W/cm^2^–500 mm/s.

Figure 6 shows the surface morphology and orthogonal profile curves of samples processed under representative Gaussian laser parameters. Compared to the as-fabricated surface with unmelted particles (Figure 1b), the laser-treated surface is considerably smoother. At 4586 W/cm^2^ and 100 mm/s, Ra values are 4.8 μm (x) and 3.2 μm (y) (Figure 6a). The low scan speed induces strong convective flow in the melt pool, producing pronounced undulations; pores along the x-direction further increase roughness. Increasing the scan speed to 500 mm/s at the same power reduces Ra to 1.6 μm (x) and 1.3 μm (y) (Figure 6b) by shortening the laser–material interaction and limiting melt pool agitation. At 3567 W/cm^2^ and 500 mm/s (Figure 6c), Ra rises to 3.9 μm (x) and 4.9 μm (y), as lower energy is insufficient to fully smooth surface asperities.

**Figure 7:**
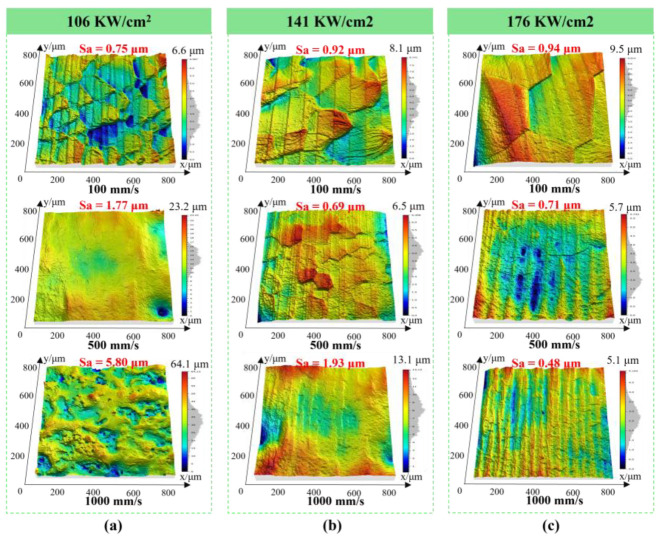
3D surface profiles of samples subjected to top-hat beam laser polishing at different conditions. The applied laser power densities were (**a**) 106 W/cm^2^, (**b**) 141 W/cm^2^, and (**c**) 176 W/cm^2^, with the scanning speed varying across the range of 100 to 1000 mm/s.

The surface topography obtained after top-hat beam laser polishing is illustrated in Figure 7. The measurements show that processing at 106 kW/cm^2^ and 100 mm/s results in a surface roughness of 0.75 μm. Increasing the scanning speed to 1000 mm/s leads to a gradual deterioration in surface smoothness. This trend is primarily due to a reduction in heat input, which becomes insufficient to fully remelt and redistribute the peaks into the adjoining valleys [29,30]. Holding the scanning speed constant at 100 mm/s and increasing the laser power density to 176 kW/cm^2^ results in minimal change in surface roughness; however, the original β grain size increases. The slight roughness increase under this condition is primarily due to uneven deformation between the original β grains [17]. At a constant power density of 176 kW/cm^2^, increasing the scanning speed from 100 mm/s to 1000 mm/s leads to finer surface grains, less intergranular deformation, and a lower roughness value of 0.48 μm.

**Figure 8:**
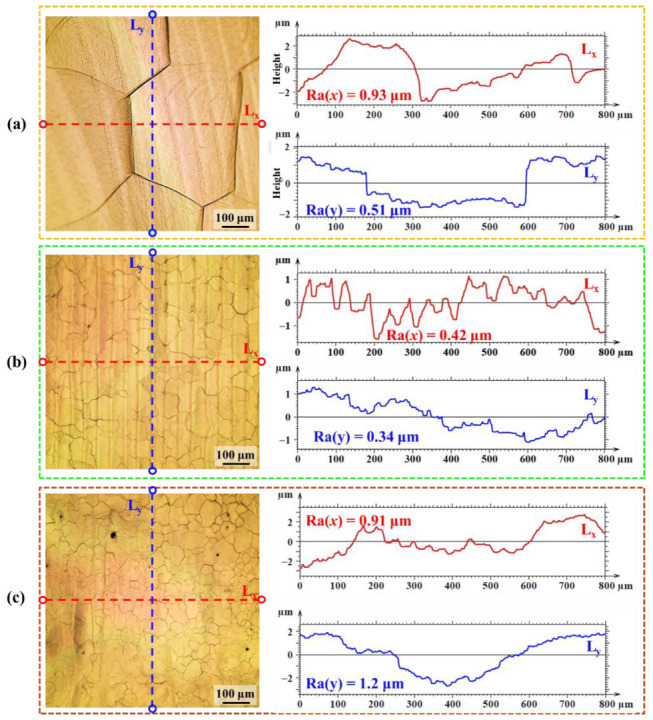
Surface morphology and profile curves of specimens processed under representative top-hat laser polishing parameters: (**a**) 176 W/cm^2^–100 mm/s, (**b**) 176 W/cm^2^–1000 mm/s, (**c**) 141 W/cm^2^–1000 mm/s.

Figure 8 shows the surface morphology and orthogonal profile curves of samples polished with a top-hat laser. At 176 W/cm^2^ and 100 mm/s (Figure 8a), Ra values are 0.9 μm (x) and 0.8 μm (y). The low scan speed promotes coarser prior-β grains, and the β→α transformation during cooling induces strain at grain boundaries [31,32], causing height irregularities and increased roughness. Increasing the scan speed to 1000 mm/s at the same power (Figure 8b) refines the prior-β grains, reducing transformation strain and lowering Ra to 0.4 μm (x) and 0.3 μm (y); the remaining height variations correspond to grain boundaries and the bulge of laser tracks [33]. At 141 W/cm^2^ and 1000 mm/s (Figure 8c), Sa rises to 0.9 μm (x) and 1.4 μm (y) due to insufficient energy to fully remelt the surface and level peaks [34].

### 4.2. Microstructure

**Figure 9:**
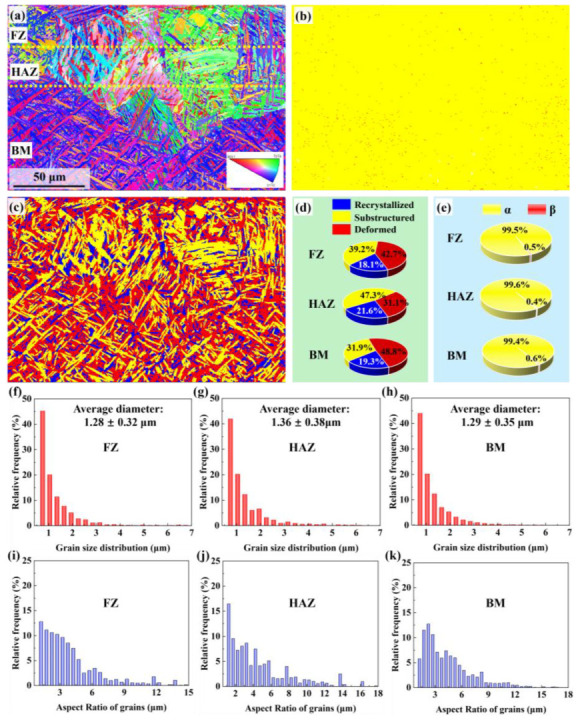
Cross-sectional EBSD results for Gaussian source-polished specimen obtained at 4586 kW/cm^2^–500 mm/s. (**a**) Inverse pole figure map. (**b**) Phase distribution. (**c**) Grain type distribution. (**d**) Grain type ratio. (**e**) Phase distribution ratio. (**f**–**h**) Grain size distribution and (**i**–**k**) grain aspect ratio distribution across FZ, HAZ, and BM.

Laser polishing strongly affects surface remelting and microstructure via thermal effects. Figure 9 presents EBSD analysis after Gaussian laser polishing. The cross-section (Figure 9a) identifies the FZ, HAZ, and BM. Phase maps (Figure 9b) show a similar composition across all regions, predominantly α-phase. In the FZ, α-phase accounts for 99.5%, with trace β, while the HAZ resembles the BM. The focused Gaussian heat input induces rapid solidification, keeping the material in the high-temperature β region briefly and transforming most prior-β into α [35]. Grain type maps (Figure 9c) show the FZ is dominated by deformed grains (42.7%), while recrystallized grains prevail in the HAZ (47.3%); deformed grains constitute 48.8% in the BM (Figure 9d). Grain size distributions (Figure 9f–h) are centered near 1 μm, with average sizes of 1.28 μm (FZ) and 1.25 μm (BM), indicating rapid cooling promotes fine grains [36]. Grain aspect ratios (Figure 9i–k) reveal predominantly acicular morphologies, with aspect ratios between 2 and 6.

**Figure 10:**
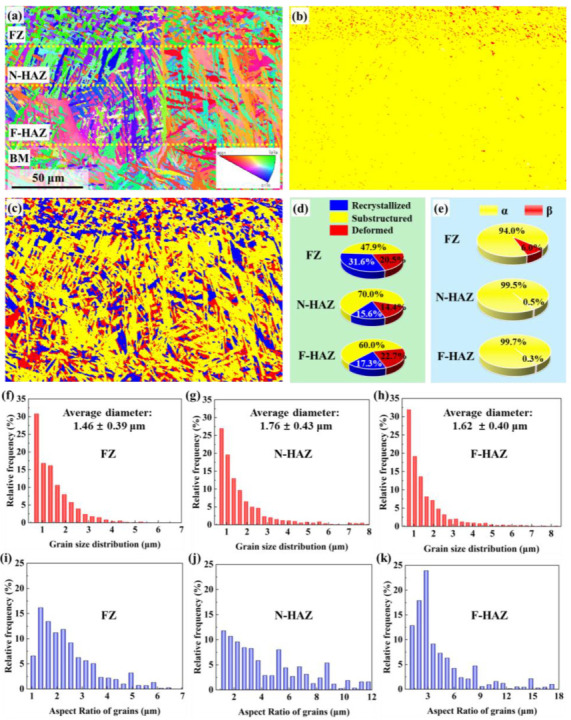
Cross-sectional EBSD characterization of sample processed under top-hat laser polishing conditions of 176 kW/cm^2^–1000 mm/s. (**a**) IPF orientation map. (**b**) Phase distribution. (**c**) Grain classification. (**d**) Grain type fraction. (**e**) Phase fraction statistics. (**f**–**h**) Grain size distributions and (**i**–**k**) grain aspect ratio distributions within the FZ and HAZ.

Figure 10 presents the EBSD analysis of the near-surface microstructure after top-hat laser polishing. The cross-section (Figure 10a) shows the FZ at the top. To enable a more detailed analysis of the microstructure in this region, the HAZ was subdivided into near-field HAZ (N-HAZ) and far-field HAZ (F-HAZ) regions based on the distance from the heat source. Phase maps (Figure 10b) reveal increased β-phase in the FZ (94% α, 6% β), while the HAZs resemble the BM, with α > 99.5%. The uniform heat input of the top-hat beam creates a shallower temperature gradient and lower cooling rate than the Gaussian process [16], prolonging dwell in the β-phase field. Grain type maps (Figure 10c) show more recrystallized (31.6% in FZ) and recovered grains (70% in N-HAZ, 60% in F-HAZ), indicating an annealing-like effect that relieves residual stresses and promotes grain recovery. Grain size distributions (Figure 10f–h) remain narrow, with the FZ averaging 1.46 μm due to thermal exposure. Grain aspect ratios (Figure 10i–k) indicate a trend toward equiaxed morphologies, primarily ranging from 1 to 3.

**Figure 11:**
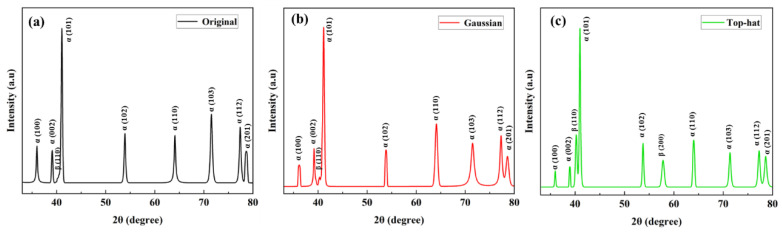
The surfaces XRD patterns of three types: (**a**) Original, (**b**) Gaussian specimen obtained at 4586 kW/cm^2^–500 mm/s, and (**c**) top-hat polishing conditions of 176 kW/cm^2^–1000 mm/s.

To further verify the phase distribution, XRD analysis was conducted on the original and laser-polished surfaces. Figure 11 shows the XRD patterns for the original surface, the Gaussian laser-polished surface, and the top-hat laser-polished surface under optimal parameters. The original surface primarily consists of hexagonal α phase with a small fraction of cubic β phase (Figure 11a). After Gaussian laser polishing, the surface remains α-phase dominant due to the high cooling gradient (Figure 11b), with only a small amount of β phase indicated by the weak (110)_β_ peak. In contrast, the top-hat laser-polished surface (Figure 11c) shows increased intensities of the β (110) and (200) peaks, indicating a higher retained β-phase fraction. This suggests that the wider and more uniform thermal exposure associated with the top-hat beam promotes a more balanced phase distribution and more uniform alloying element distribution in the polished layer.

**Figure 12:**
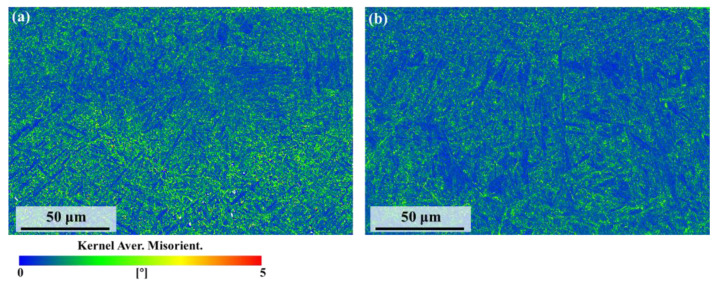
Kernel average misorientation maps of the sample under (**a**) Gaussian and (**b**) top-hat laser polishing.

Figure 12 shows the kernel average misorientation (KAM) maps after polishing. Figure 12a presents the KAM map after Gaussian laser polishing, where the overall values are relatively high, with most regions appearing green and corresponding to values around 2.5°. Both the melted zone and the substrate region exhibit relatively high KAM values. In contrast, after top-hat laser polishing (Figure 12b), the overall KAM values decrease. In the melted zone, blue and green regions are more uniformly distributed, while in the heat-affected zone, grain growth of the α phase and stress release lead to lower KAM values, with some α grains appearing blue, indicating reduced local misorientation.

**Figure 13:**
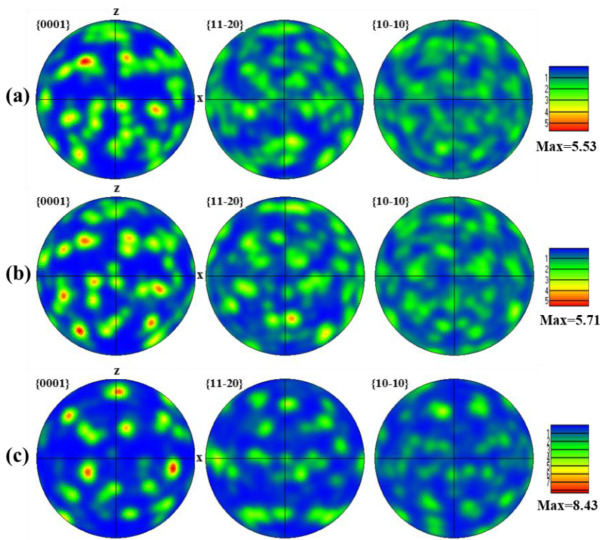
α-phase pole figures of the GS specimen in the (**a**) FZ, (**b**) HAZ, and (**c**) BM regions.

Figure 13 shows the α-phase texture of the Gaussian laser-polished specimen via standard pole figures. In the FZ (Figure 13a), α grains are widely dispersed, forming acicular α/α′ structures due to rapid β → α transformation and extensive nucleation, resulting in a weak texture intensity of 5.53 [37]. In the HAZ (Figure 13b), sub-melting thermal input promotes partial recrystallization and grain growth, slightly aligning orientations and increasing the texture intensity to 5.71. The BM (Figure 13c) exhibits the highest texture intensity of 8.43, with coarser α grains and a strong preferential orientation, as indicated by the concentrated blue and purple regions in the IPF map (Figure 9a).

**Figure 14:**
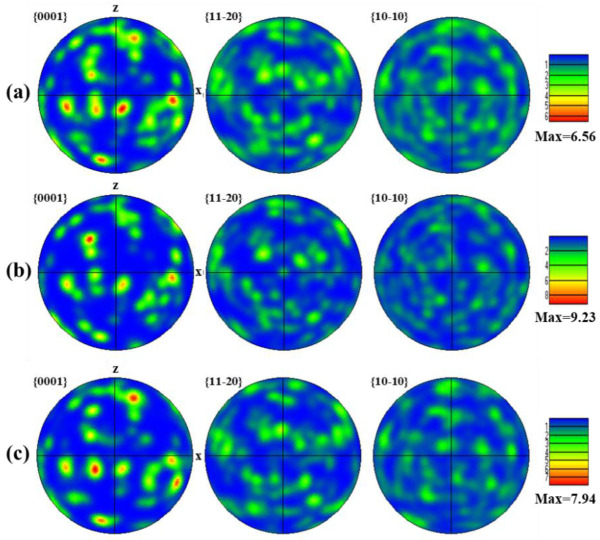
α-phase pole figures of the top-hat specimen in the (**a**) FZ, (**b**) N-HAZ, and (**c**) F-HAZ regions.

Figure 14 shows the α-phase texture intensity of the top-hat laser-polished surface via standard pole figures. In the FZ (Figure 14a), the texture intensity is 6.56. The larger top-hat spot diameter prolongs dwell time, promoting formation of equiaxed α grains with a more uniform orientation [38]. Although higher than the Gaussian FZ, this intensity remains below that of the BM. In the N-HAZ, thermal exposure causes α-grain growth, producing a more concentrated orientation. In contrast, the FHAZ experiences weaker thermal effects, limiting grain growth; partial recrystallization alters orientations, resulting in a texture intensity of 7.94. The texture intensities reported in this study reflect the degree of anisotropy in the material. Stronger textures, characterized by higher intensity values, indicate more pronounced anisotropy. Moreover, phase transformations during processing are often texture-dependent, as certain crystal orientations may be more or less prone to undergoing specific transformations. For instance, the development of a particular texture in the material can influence the formation of secondary phases or grain growth patterns.

## 5. Discussion

### 5.1. Evolution Mechanisms of the Surface Topography

**Figure 15:**
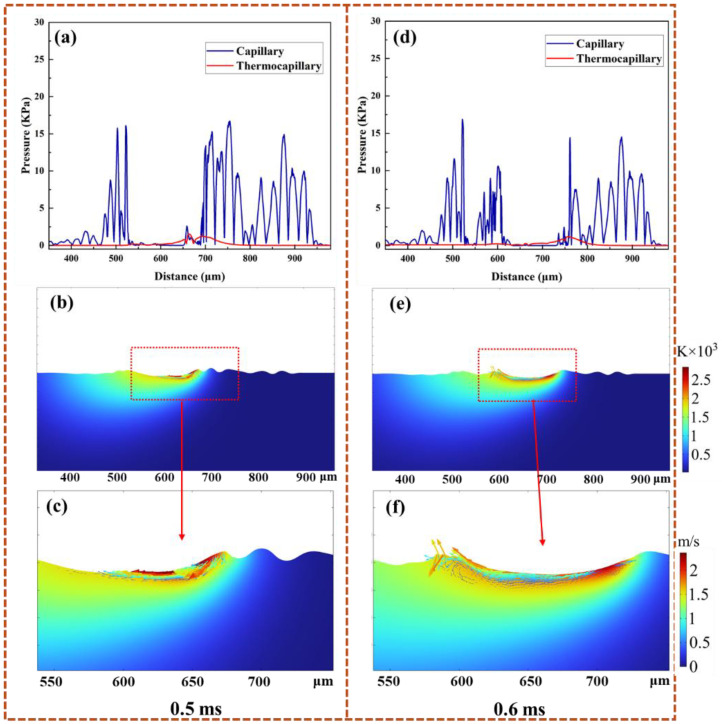
Flow dynamics in the molten pool for the GS process at 0.5 ms and 0.6 ms. (**a**,**d**) Capillary and thermocapillary force distributions. (**b**,**e**) Velocity and temperature field evolution at the respective times. (**c**,**f**) Enlarged images of the relevant areas in (**b**,**e**).

To investigate the laser polishing mechanisms in additively manufactured Ti-6Al-4V, COMSOL Multiphysics 6.9 simulations were performed for both Gaussian (4586 W/cm^2^, 500 mm/s) and top-hat (176 W/cm^2^, 1000 mm/s) laser conditions. Figure 15 illustrates the simulated melt pool thermal–fluid behavior during Gaussian laser polishing at 0.5 and 0.6 ms. At 0.5 ms (Figure 15a–c), the melt pool center exceeds 2600 K with fluid velocities above 2.2 m/s. The high temperature lowers surface tension at the center, while steep gradients generate strong Marangoni forces that drive molten material from peaks opposite to the scanning direction [39,40]. At 0.6 ms (Figure 15d–f), the advancing laser induces rearward flow at the leading edge, forming a trailing-edge peak and residual surface height differences that contribute to roughness [41,42].

**Figure 16:**
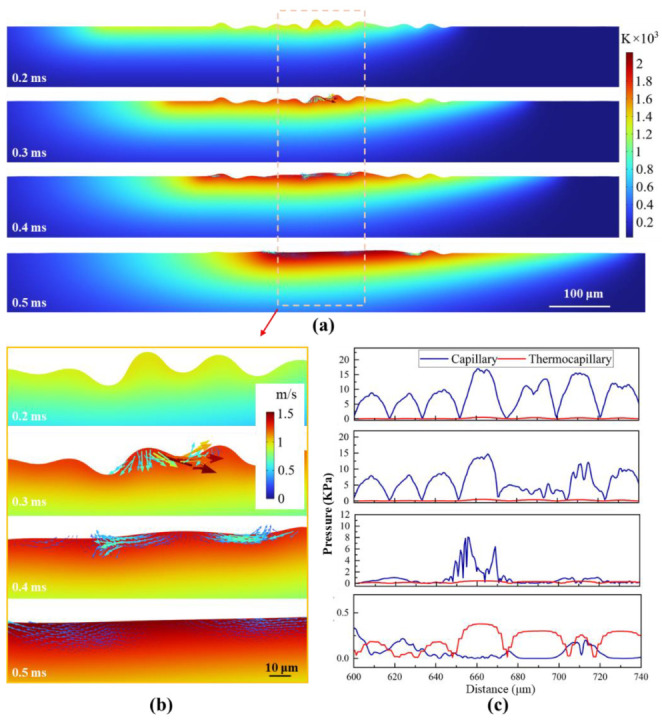
Molten pool dynamics under top-hat laser processing. (**a**) Time-dependent changes in the temperature field. (**b**) Magnified view of the temperature and velocity fields from (**a**) (The arrows represent the velocity vector). (**c**) Temporal variation in the capillary and thermocapillary forces.

Figure 16 illustrates the simulated melt pool behavior during top-hat laser polishing at 0.2–0.5 ms. At 0.3 ms, the surface reaches the melting point, liquefying asperities. Reduced surface tension, combined with gravitational, capillary, and Marangoni forces, drives molten metal into nearby valleys. The diffuse temperature field produces a moderate thermal gradient, yielding a peak flow velocity below 1.5 m/s. By 0.4 ms, surface peaks further melt and flow, reducing elevation differences and capillary pressure gradients, which lowers the peak velocity to ~0.6 m/s. At 0.5 ms, surface irregularities are minimized, the surface is fully molten, Marangoni forces drop to ~0.2 kPa, and flow becomes predominantly laminar. This controlled melt flow contributes to the enhanced surface smoothness of the titanium alloy [18].

**Figure 17:**
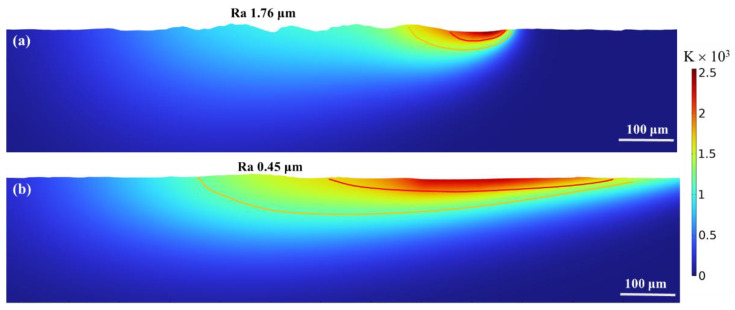
Surface topography after polishing: (**a**) Gaussian process, (**b**) top-hat process.

The polishing results were compared to the previously analyzed surface morphology and thermal distribution to confirm consistency. Figure 17 shows the post-polishing surface and laser-heated regions. After Gaussian laser polishing, the surface roughness reached 1.76 μm, consistent with experimental measurements (Figure 6b). In the melt pool, backward flow driven by surface tension produced a width of ~50 μm and a depth of ~30 μm, while the heat-affected zone (HAZ) extended ~25 μm, aligning with Figure 9a. As shown in Figure 17b, the surface roughness after top-hat laser polishing was approximately 0.45 μm, which is close to the surface roughness obtained from the earlier experimental measurements (Figure 8b). The melting depth during top-hat laser polishing was approximately 25 μm. Because of the larger spot diameter, the heat input was more dispersed, resulting in a larger heat-affected zone with a depth close to 60 μm. Therefore, for a more detailed analysis, the heat-affected zone in Figure 10a was divided into a near-field heat-affected zone and a far-field heat-affected zone.

### 5.2. Microstructure Evolution Mechanisms

**Figure 18:**
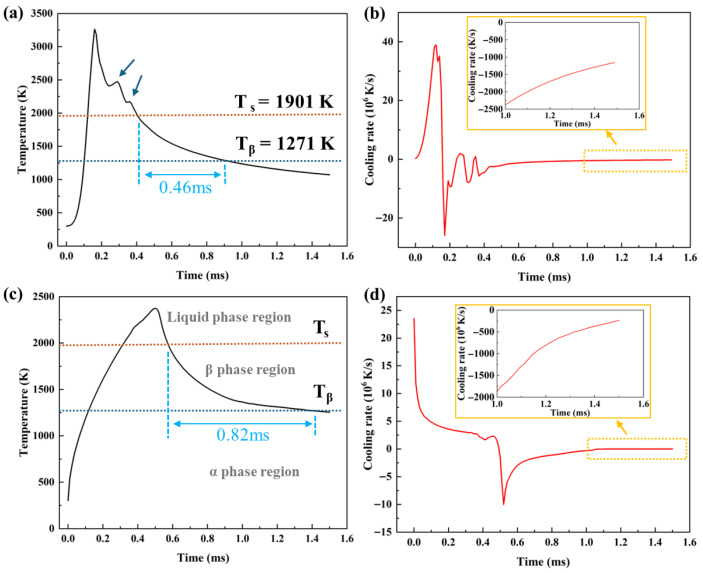
Thermal history cycles for Gaussian and top-hat laser polishing on the surface presented in Figure 2. (**a**,**b**) Temperature–time and heating/cooling rate–time curves measured at point (500, 0) on the Gaussian-polished surface. (**c**,**d**) Temperature–time and heating/cooling rate–time curves measured at point (600, 0) on the top-hat-polished surface.

Surface remelting during laser polishing produces variations in temperature and cooling rate that strongly influence microstructure [19]. To elucidate these effects, surface temperature profiles were extracted via simulation and correlated with observed microstructural features. Figure 18 shows the temperature and cooling rate evolution for both laser processes. During Gaussian laser polishing, the melt pool temperature rises sharply at 40 × 10^6^ K/s, exceeding 3000 K at the center, and cools at a maximum rate of 28 × 10^6^ K/s. This rapid thermal cycle promotes fine-grain formation and higher residual stress [43]. Notably, post-laser passage, the temperature exhibits two successive increases due to the melt pool wake effect (Figure 14) [41]. In contrast, top-hat laser polishing shows a slower heating rate of 22 × 10^6^ K/s. The larger spot diameter extends dwell time in the β-transus region, enhancing β-phase retention [44]. The melt pool subsequently cools at 11 × 10^6^ K/s, favoring grain growth and a transition toward a more equiaxed morphology.

**Figure 19:**
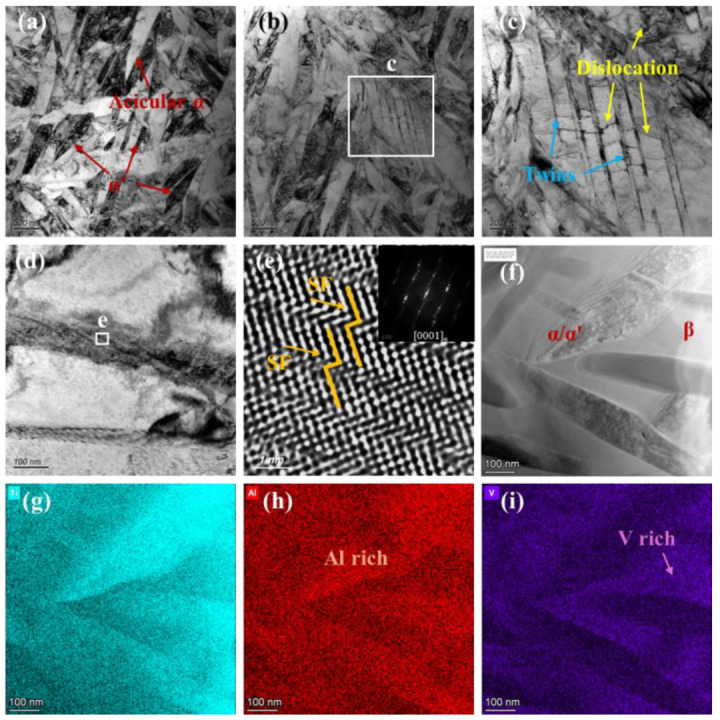
TEM observation results of the FZ for the GS specimen. (**a**–**d**) Bright-field image of microstructure. (**e**) HRTEM image of stacking faults (SF) with FFT. (**f**–**i**) HAADF imaging and corresponding EDS maps.

**Figure 20:**
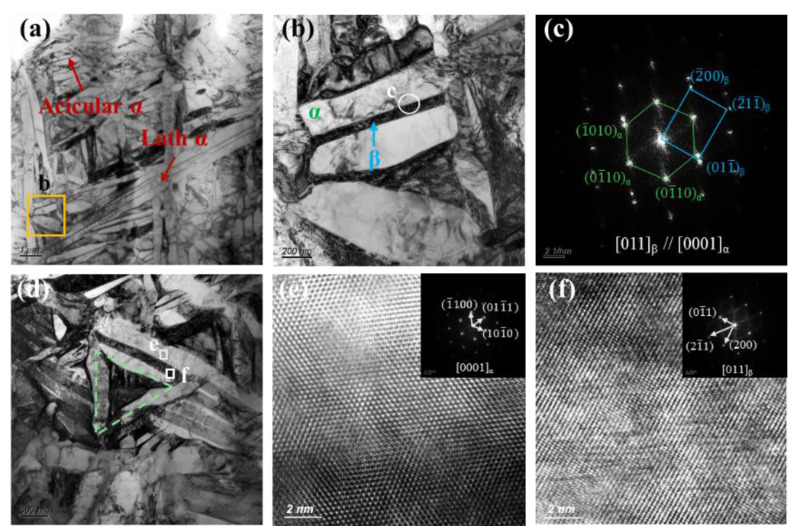
TEM observation results of the HAZ for the GS specimen. (**a**,**b**) Bright-field image of microstructure. (**c**) SAED pattern from the area marked by the white circle in (**b**). (**d**) Triangular structure cluster microstructure. (**e**,**f**) HRTEM image and the corresponding FFT pattern.

To further examine microstructural evolution, TEM analysis of the Gaussian-polished specimen was performed. In the FZ (Figure 19a), the microstructure is dominated by acicular α and α′ phases. High densities of twin boundaries and dislocations are observed within and around these phases (Figure 19c), indicating substantial internal stresses induced by the rapid cooling of the GS process. Twinning initiates at subgrain boundaries when local stress exceeds a critical level, with partial dislocations extending into the grain interior. These stresses also promote stacking fault formation, evident at α/α′ phase edges (Figure 19d), with HRTEM confirming atomic-scale misalignment between layers (Figure 19e). HAADF imaging and elemental mapping (Figure 19f–i) show Al enrichment in the acicular α/α′ phases and V enrichment in residual β at grain boundaries. In the HAZ, the microstructure consists of acicular and plate-like α phases separated by thin β layers (Figure 20b). SAED patterns (Figure 20c) confirm that the α plates and β layers maintain the Burgers orientation relationship, (011)β//(0001)α, indicating complete α transformation during thermal cycling [45]. Partially developed triangular basket-weave α structures are observed (Figure 19d), with HRTEM and FFT analysis (Figure 20e,f) verifying that α plates and surrounding β phases preserve the Burgers orientation relationship.

**Figure 21:**
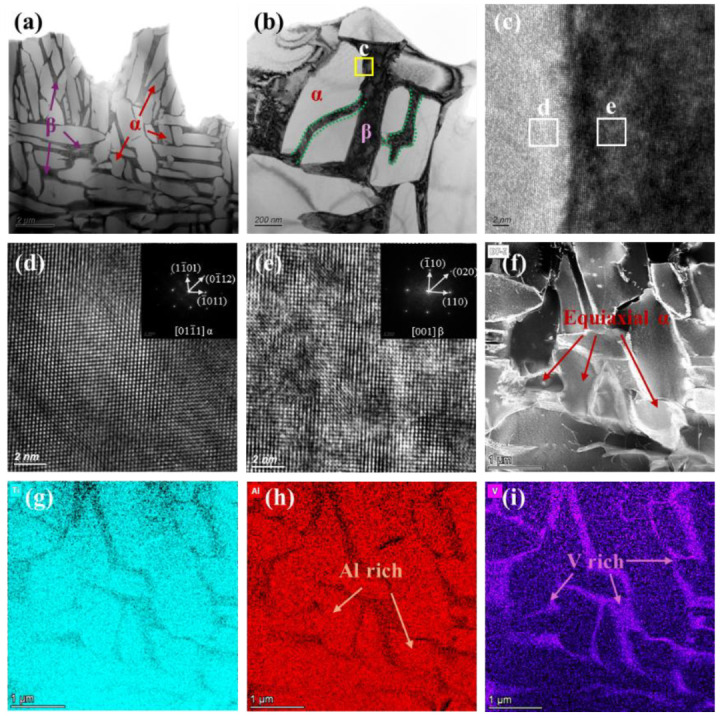
TEM observation results of the FZ for the Top-hat specimen. (**a**,**b**) Bright-field image of microstructure. (**c**) HRTEM image. (**d**,**e**) HRTEM image and the corresponding FFT pattern from white circle marked in (**c**). (**f**–**i**) Dark-field (DF) image and the EDS mapping.

The top-hat beam, with its broader spot and uniform energy distribution, promotes complete recrystallization of surface grains and extended elemental diffusion. TEM analysis of the FZ (Figure 21) shows α phase with β phase along grain boundaries (Figure 21a). The β phase grows inward from the α boundaries, subdividing the α matrix into discrete, nearly equiaxed subgrains (Figure 21b). After the laser heat source passes, the temperature drops below the melting point and β grains begin to precipitate from the liquid. The dispersed heat input of the top-hat laser extends the dwell time in the high-temperature β-phase field (~0.82 ms, Figure 18c), facilitating greater retention of β phase through enhanced diffusion of β-stabilizing elements. As the temperature drops below the melting point and then the β transus, β transforms partially into α, while the remaining β diffuses along α/β boundaries, fragmenting α plates and promoting a more equiaxed morphology (Figure 21c) [46]. The α/β interface is sharp, with reduced defects after sufficient recrystallization. HRTEM and FFT analyses (Figure 21d,e) show that the α and β phases no longer strictly follow the Burgers orientation relationship, with the α phase orientations altered post-recrystallization. The pronounced propagation of β along α grain boundaries and its intrusion into grain interiors ultimately produces a high density of equiaxed α grains (Figure 21f–i).

**Figure 22:**
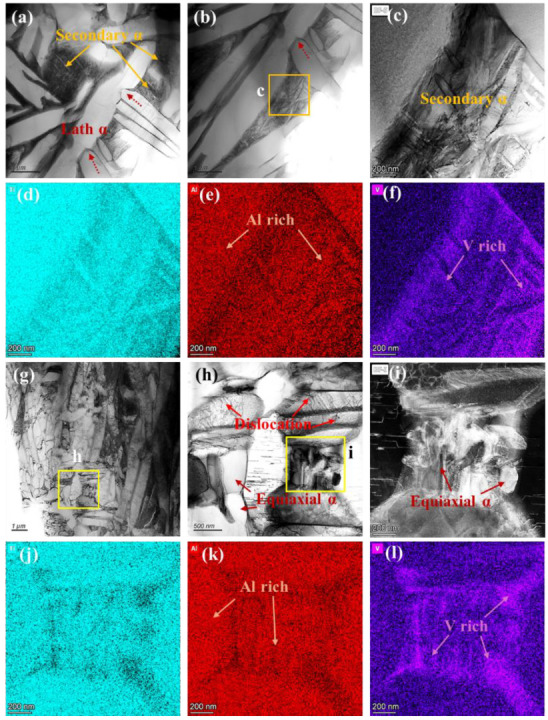
TEM observation results of the HAZ for the top-hat specimen. (**a**,**b**) Bright-field image of microstructure in N-HAZ. (**c**–**f**) Bright-field (BF) image and the corresponding EDS mapping in N-HAZ. (**g**,**h**) Bright-field image of microstructure in F-HAZ. (**i**–**l**) Dark-field (DF) image and the corresponding EDS mapping in F-HAZ.

Figure 22 presents TEM micrographs of the HAZ in the top-hat-polished specimen. In the N-HAZ, the microstructure primarily consists of plate-like α and secondary α phases (Figure 22a,b), with red dashed arrows indicating partial fragmentation of the α plates. However, the lower temperature compared to the FZ limits elemental diffusion at defect sites, preventing complete fragmentation. Figure 22c shows significant secondary α precipitation within triangular regions of the α plates. During SLM, thermomechanical coupling induces substantial stress and stored energy at defect sites [47], which may influence subsequent microstructural evolution. As the top-hat laser traverses the surface, the heat-affected zone experiences a temperature gradient from the β-phase region near the melt pool to the α-phase region below. Under these conditions, the material may briefly approach the β-phase field, while predominantly remaining within the α-phase region below T_β_. As shown in Figure 18d, the cooling rate near the melt pool drops below 200 K/s once T_β_ is crossed, which may extend the residence time within a subcritical temperature range. This thermal condition is likely consistent with a subcritical heat-treatment-like effect, which could promote the precipitation of secondary phases. In this context, the formation of secondary α may be associated with the evolution of V-enriched metastable β into secondary α and retained β [48]. Figure 22d–f show that secondary α appears to nucleate in the V-rich triangular regions and grows outward along the α plate boundaries.

In the F-HAZ (Figure 22g–i), finer grains are observed, which may be related to the relatively lower thermal input that limits α coarsening. A high density of dislocations is retained within the grains (Figure 22h), while additional fine equiaxed α grains precipitate over time. Figure 22j–l indicate that these fine α grains are often enriched in V, suggesting that low-temperature laser exposure may contribute to the decomposition of supersaturated martensite into α and metastable β phases [49]. The subsequent evolution of these V-enriched regions may further contribute to the formation of fine equiaxed α grains and β along grain boundaries.

## 6. Conclusions

Ti-6Al-4V components fabricated via SLM typically exhibit high surface roughness, limiting their direct applicability. This study demonstrates that top-hat laser polishing effectively enhances the surface quality of SLM-produced Ti-6Al-4V. Through a comparative investigation with Gaussian laser polishing, the underlying melt pool dynamics and microstructural evolution mechanisms responsible for the superior surface finish of top-hat laser-polished surfaces are elucidated. The main conclusions of this work are summarized as follows.

1. The top-hat laser polishing technique employs a larger beam diameter with a uniform energy profile, which substantially enhances surface finish. The titanium alloy processed at 176 kW/cm^2^ and 1000 mm/s exhibited a minimum Sa of 0.48 μm. This outcome corresponds to a 95.3% reduction in surface roughness relative to the original surface.
2. Gaussian laser polishing generates stronger convection within the melt pool due to pronounced Marangoni forces and wake flow effects, which collectively increase surface roughness. The top-hat laser, however, achieves superior surface quality through its distinct melt pool dynamics: molten material from multiple peaks fills surface valleys, while long-range horizontal flow promotes uniform leveling, resulting in effective surface smoothing.
3. The fusion zone from top-hat laser polishing primarily consists of equiaxed α grains and 6% β phase. In the heat-affected zone, the microstructure probably underwent a subcritical heat-treatment-like condition, which may be associated with partial martensite decomposition during subsequent laser passes, forming lath-like structures and secondary α phases.
4. Additionally, top-hat laser polishing has the potential to reduce the surface stress induced by rapid cooling during the SLM fabrication process. Specifically, the proportion of recrystallized grains in the fusion zone increases from 19.2% to 39.2%, while that of deformed grains decreases from 48.8% to 20.6%.

## Figures and Tables

**Table 1:** Summary of operational parameters for laser polishing processes.

Process Parameters	Symbol	GS	Top-Hat
Laser power density (kW/cm^2^)	E	1528–4586	71–176
Laser power (W)	P	30–90	100–500
Laser scanning velocity (mm/s)	*Vs*	100–1500	100–1500
Spot diameter (μm)	*D*	50	600
Scan track spacing (μm)	Δy	5	60
Scan overlap percentage (%)	*k*	90	90

**Table 2:** Temperature-sensitive material properties of Ti-6Al-4V incorporated into the simulation [21,22].

Physical Property	Symbol	Value
Temperature of solid phase (K)	$T_{s}$	1880
Temperature of liquid phase (K)	$T_{l}$	1922
Melting temperature (K)	$T_{m}$	1901
Temperature of the surroundings (K)	$T_{0}$	293.15
Solid-phase density (kg·m^−3^)	$\rho_{s}$	4430
Liquid-phase density (kg·m^−3^)	$\rho_{l}$	4010
Specific heat of solid	$C_{s}$	671
Specific heat of liquid	$C_{l}$	830
Latent heat of fusion (J kg^−1^)	$L_{m}$	2.86 × 10^5^
Emissivity	ϵ	0.6
Laser absorption coefficient	α	0.33
Thermal expansion coefficient (K^−1^)	β	1.12 × 10^−5^
Heat transfer coefficient (W·m^−2^·K^−1^)	h	10
Surface tension of pure titanium (N m^−1^)	$\gamma_{m}$	1.59
Constant in surface tension gradient (N m^−1^ K^−1^)	$A_{\gamma }$	2.81 × 10^−4^
Atmospheric pressure (Pa)	Atm	1.013 × 10^5^

**Table 3:** Key laser polishing parameters implemented in simulation.

Process Parameters	Symbol	GS	Top-Hat
Energy input density of laser (kW/cm^2^)	*I*	4586	176
Scanning speed (mm/s)	*Vs*	500	1000
Laser beam diameter (μm)	*D*	45	600

**Table 4:** A summary of boundary conditions for the physical domains.

Boundary Condition	Physical Condition	Boundary
Thermal energy transfer	Laser radiation	1
Free deformation	Free surface	1
Convection	Natural convection	1, 2, 3
Diffuse surface	Thermal radiation	1, 2, 3
No-slip wall	Wall	2, 3, 4
Thermal insulation	Insulation	4

## Data Availability

The original contributions presented in this study are included in the article. Further inquiries can be directed to the corresponding author.

## Supplementary Materials

The following supporting information can be downloaded at: https://www.mdpi.com/article/10.3390/nano16090505/s1. Table S1: Comparative analysis of additional normalized energy input parameters. Table S2: Comparative analysis of the effects of different mesh sizes on the molten pool. Figure S1: Different average mesh size discretizations in the numerical model: (a) 3 μm, (b) 4 μm, (c) 5 μm. Figure S2: Molten pool morphology at 0.5 ms under different average mesh sizes. Table S3: Comparative analysis of the effects of different time intervals on the molten pool. Figure S3: Molten pool morphology at 0.5 ms under different simulation time intervals.
