# Supplementary material for: Surface Quality Enhancement of SLM-Fabricated Ti-6Al-4V via Top-Hat Laser Polishing: Melt Pool Dynamics and Microstructural Evolution
Source: Nanomaterials (Basel). 2026 Apr 22;16(9):505. doi: 10.3390/nano16090505 (PMC13164693; doi:10.3390/nano16090505)
Supplement: Supplementary file 1 [file nanomaterials-16-00505-s001.zip › nanomaterials-4238873-supplementary.pdf]

Supplementary Materials

The calculated energy metrics indicate that, although the absolute power density differs between the Gaussian and top-hat systems, the normalized parameters such as line energy and interaction time fall within comparable ranges for the selected processing conditions. However, despite these similarities, distinct differences in melt pool morphology and surface quality are still observed. This suggests that, beyond the total energy input, the spatial distribution of energy governed by the beam profile plays a significant role in determining the thermal response and microstructural evolution.

To enable a more rigorous comparison between Gaussian and top-hat polishing conditions, additional normalized energy input parameters were evaluated, including line energy E_l, areal energy density E_a, interaction time Δt, and overlap-normalized heat input E_{OV}.

$$E_l = \frac{P}{V}\ ,\ E_a = \frac{P}{V \cdot D}\ ,\ \Delta t = \frac{D}{V}\ ,\ E_{OV} = \frac{P}{V \cdot \Delta y} \quad \text{(S1)}$$

As shown in Table S1, significant differences exist in the absolute laser parameters due to variations in spot size and power. However, certain normalized parameters fall within partially overlapping ranges. For example, when maintaining the same speed at 500 mm/s, the areal energy density for Gaussian polishing (1.2–3.6 J/mm^2) overlaps with that of the top-hat beam (0.67–1.67 J/mm^2), particularly at intermediate power levels. Similarly, overlap-normalized heat input shows comparable magnitudes between the two systems under selected conditions. In contrast, the interaction time differs significantly, with the top-hat beam exhibiting a much longer interaction time due to its larger spot size.

Furthermore, when the laser power is kept constant, the areal energy density and overlap-normalized heat input exhibit comparable ranges at intermediate scanning speeds. However, a substantial difference is observed in interaction time, with the top-hat beam exhibiting values approximately one order of magnitude higher due to its larger spot size. These results indicate that the two polishing modes operate under distinct thermal regimes. The Gaussian beam is characterized by high energy

concentration and short interaction time, while the top-hat beam provides a more spatially uniform energy input with significantly longer thermal interaction.

This suggests that the differences in surface morphology and microstructural evolution may arise from a combined effect of energy input, interaction time, and thermal history, within which the beam profile plays a critical role by governing the spatial distribution of energy and the resulting thermal gradients.

Table S1. Comparative analysis of additional normalized energy input parameters.

Beam	Power (W)	Speed (mm/s)	Power density (KW/cm^2)	Line energy (J/mm)	Areal energy (J/mm^2)	Interaction time (ms)	Overlap heat input (J/mm^2)
GS	30	500	1528	0.06	1.20	0.10	12
GS	50	500	2548	0.10	2.00	0.10	20
GS	70	500	3567	0.14	2.80	0.10	28
GS	90	500	4586	0.18	3.60	0.10	36
GS	90	100	4586	0.90	18.00	0.50	180
GS	90	200	4586	0.45	9.00	0.25	90
GS	90	1000	4586	0.09	1.80	0.05	18
GS	90	1500	4586	0.06	1.20	0.033	12
Top-hat	200	500	71	0.40	0.67	1.20	6.67
Top-hat	300	500	106	0.60	1.00	1.20	10.00
Top-hat	400	500	141	0.80	1.33	1.20	13.33
Top-hat	500	500	176	1.00	1.67	1.20	16.67
Top-hat	500	100	176	5.00	8.33	6.00	83.33
Top-hat	500	200	176	2.50	4.17	3.00	41.67
Top-hat	500	1000	176	0.50	0.83	0.60	8.33
Top-hat	500	1500	176	0.33	0.56	0.40	5.56

To ensure numerical accuracy, a mesh sensitivity analysis was performed using three different mesh resolutions (fine, medium, and coarse). Figure S1 illustrates the mesh configurations with average element sizes of 3 μm, 4 μm, and 5 μm. Figure S2 presents the simulated melt pool morphology and temperature distributions for each mesh size, while Table S2 summarizes the corresponding melt pool depths and

temperature data. The characteristic melt pool depth and peak temperature were selected as representative output variables. The difference between the fine and medium mesh is less than 3%, indicating that the fine mesh provides a good balance between accuracy and computational efficiency and is therefore adopted in this study.

Table S2. Comparative analysis of the effects of different mesh sizes on the molten pool.

Mesh	Average mesh size (μm)	Melt depth (μm)	Peak temperature (K)
Fine	3	25.3	2181.5
Medium	4	25.9	2196.3
Coarse	5	25.2	2180.2

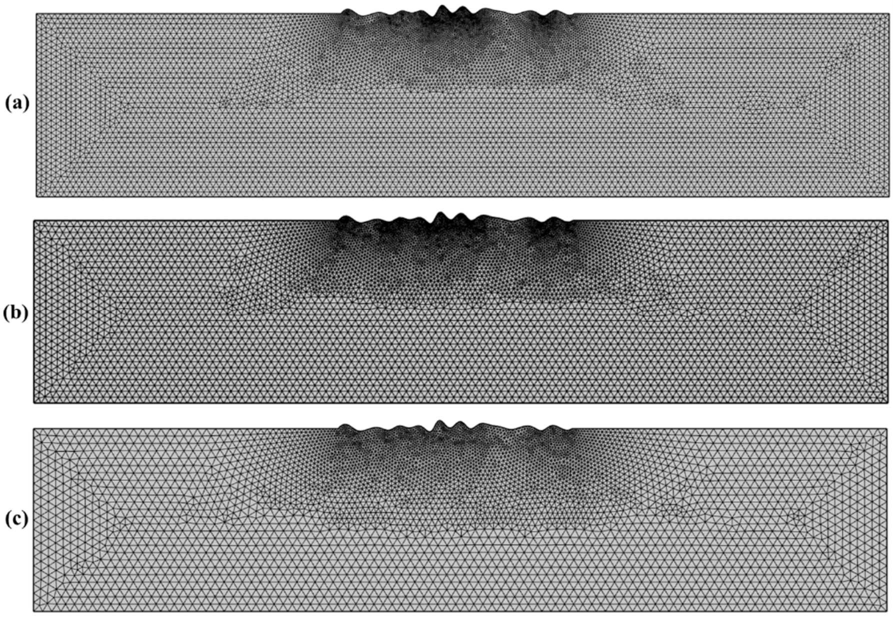

Figure S1. Different average mesh size discretizations in the numerical model: (a) 3 μm, (b) 4 μm, (c) 5 μm.

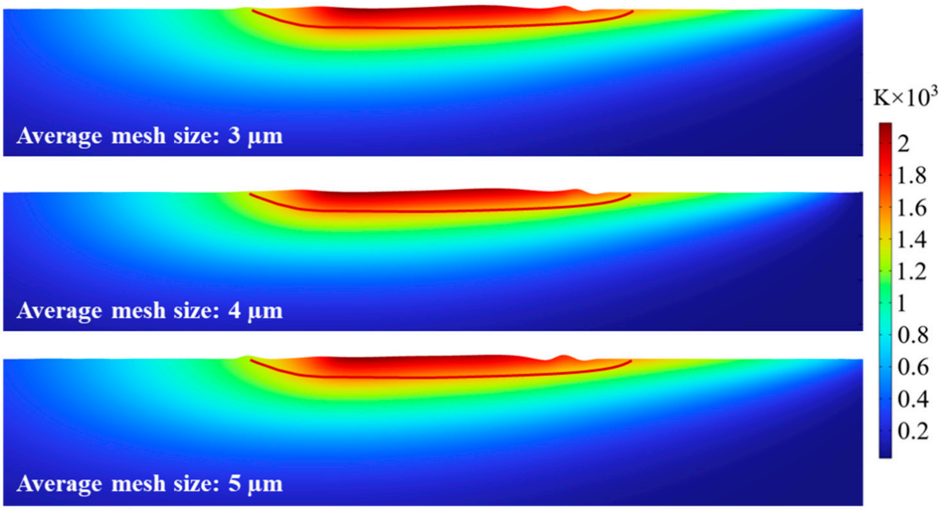

Figure S2. Molten pool morphology at 0.5 ms under different average mesh sizes.

Figure S3 shows the melt pool morphology and temperature distributions obtained using different simulation time steps. Table S3 summarizes the corresponding melt pool depths and peak temperatures. The results indicate that variations in melt pool depth and temperature are minimal across different time steps; therefore, a smaller time step of 0.01 ms was selected for the simulations in this study.

Table S3. Comparative analysis of the effects of different time intervals on the molten pool.

Time step (ms)	Melt depth (μm)	Peak temperature (K)
0.01	25.3	2181.5
0.02	25.8	2192.7
0.05	25.7	2189.8

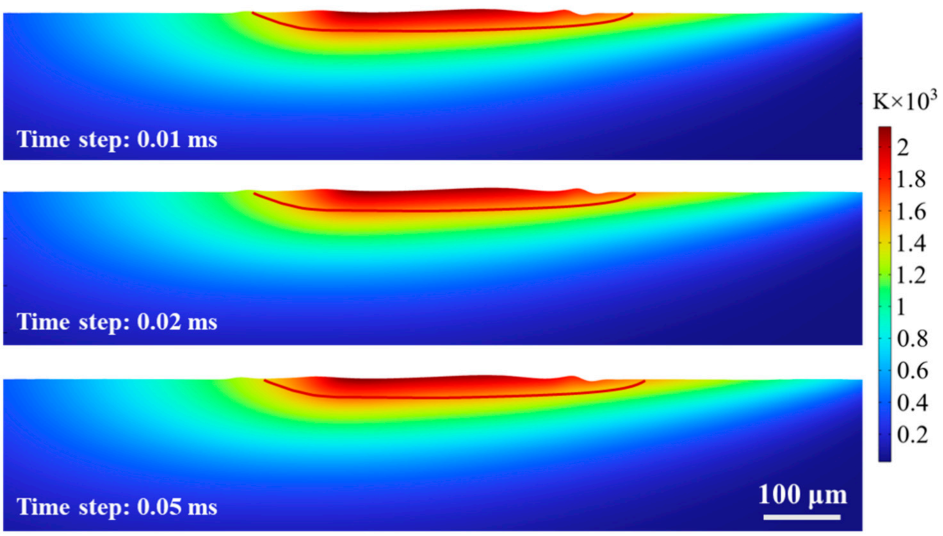

Figure S3. Molten pool morphology at 0.5 ms under different simulation time intervals.
